# Structuring and validating a prognostic model for low-grade gliomas based on the genes for plasma membrane tension

**DOI:** 10.3389/fneur.2022.1024869

**Published:** 2022-11-03

**Authors:** Jia Li, Fangfang Lv, Ting Jin

**Affiliations:** ^1^Department of Neurology, The First Affiliated Hospital of Wenzhou Medical University, Wenzhou, China; ^2^Department of Pediatric Pulmonology, The Second Affiliated Hospital and Yuying Children's Hospital, Wenzhou Medical University, Wenzhou, China; ^3^Operating Room, The First Affiliated Hospital of Wenzhou Medical University, Wenzhou, China

**Keywords:** LGG, plasma membrane tension, SH3GL2, survival, prognostic model

## Abstract

**Background:**

Recent studies indicate that cell mechanics are associated with malignancy through its impact on cell migration and adhesion. Gliomas are the most common primary malignant brain tumors. Low-grade gliomas (LGGs) include diffuse LGGs (WHO grade II) and intermediate-grade gliomas (WHO grade III). Few studies have focused on membrane tension in LGGs. Herein, we assessed the prognostic value of plasma membrane tension-related genes (MTRGs) in LGGs.

**Methods:**

We selected plasma MTRGs identified in previous studies for analysis. Based on LGG RNA sequencing (RNA-seq) data in The Cancer Genome Atlas, a prognostic signature containing four genes was constructed *via* log-rank testing, LASSO regression and stepwise multivariate Cox regression and was validated with other datasets. Additionally, functional annotation, pathway enrichment and immune and molecular characteristics of the prognostic model defined subgroups were analyzed. Thereafter, a predictive nomogram that integrated baseline characteristics was constructed to determine the 3, 5, and 10-year overall survival (OS) of patients with LGG. Differentially expressed genes were confirmed *via* quantitative reverse-transcription polymerase chain reaction (qRT-PCR) and immunohistochemistry (IHC).

**Results:**

Our MTRG prognostic signature was based on ARFIP2, PICK1, SH3GL2, and SRGAP3 expression levels. The high-risk group was more positively associated with apoptosis and cell adhesion pathways and exhibited a low IDH1 mutation rate, high TP53 mutation rate and a low 1p19q co-deletion rate. The high-risk group also exhibited incremental infiltration of immune cells, more forceful immune activities and high expression of immune checkpoints as well as benefited less from immune therapy compared with the low-risk group. Our prognostic model had better forecasting ability than other scoring systems. We found that the nomogram was a better tool for predicting outcomes for patients with LGG. Finally, qRT-PCR confirmed that SH3GL2 and SRGAP3 expression levels in glioma tissues were significantly lower than those in normal brain tissues. The results of IHC analysis confirmed that SH3GL2 protein expression was higher in patients with longer survival.

**Conclusion:**

Our plasma membrane tension-related gene prognostic signature is a prospective tool that can differentiate between prognosis, gene mutation landscape, immune microenvironment, immune infiltration and immunotherapeutic efficacy in LGG.

## Introduction

Glioma is the most common primary brain tumor that accounts for approximately 80% of all malignant brain tumors (1). Gliomas are defined as brain tumors of glial origin; depending on their malignant behavior, gliomas can be categorized into WHO grades I–IV. The most common histological types of gliomas include astrocytomas (grades I–III), oligodendrogliomas (grades II–III), oligoastrocytomas (grades II–III) and glioblastomas (grade IV) (2). The prognosis of patients with gliomas varies with the molecular subtype. compared with isocitrate dehydrogenase (IDH) wild-type glioma, mutations in IDH1/2 constitute a glioma subtype with better prognosis and distinct ontogeny. O6 methylguanine-DNA methyltransferase (MGMT) expression is repressed by MGMT promoter methylation, which results in TMZ resistance. The 1p19q co-deletion is strongly associated with an oligodendroglial phenotype and favorable prognosis (2–4). In addition, the cIMPACT-NOW update 3 recommends that IDH wild-type LGG harboring EGFR gene amplifications, chromosome 7 gain and chromosome 10 loss, or TERT promoter mutations will follow an invasive clinical course similar to IDH wild-type glioblastoma and patients with this condition have significantly shorter survival (5). LGG molecular subtyping may provide additional prognosis information regarding patient outcome; however, new risk-stratification biomarkers need to be identified.

Cell membrane tension affects various vital biological processes, including cytokinesis, cell motility, endocytosis, exocytosis and organelle function sustainability, which is closely correlated with rapid disease progression (6). Recent studies have shown that high plasma membrane tension effectively inhibits cancer cell migration and invasion by counteracting the membrane curvature sensing/production of BAR family proteins (7–9). Membrane tension regulates the cellular pluripotent state via endocytosis-mediated ERK signaling and regulates cellular metabolism through proteasome degradation of the rate-limiting metabolic enzyme phosphofructokinase to maintain high glycolytic rates in cancer cells (10, 11). Recent studies have reported the association between plasma membrane tension and tumor invasion, pluripotency and metabolism (12–14). However, there are only few studies on its association with LGG prognostic value. To better predict survival of patients with LGG, our prognostic nomogram took into account patient age, grade, IDH mutation and 1p19q co-deletion state while calculating baseline characteristics and risk scores. Our results indicate that our plasma membrane tension-related prognostic model may be a potential prognostic indicator and therapeutic target for LGG.

## Materials and methods

### Study flow diagram

**Figure 1:**
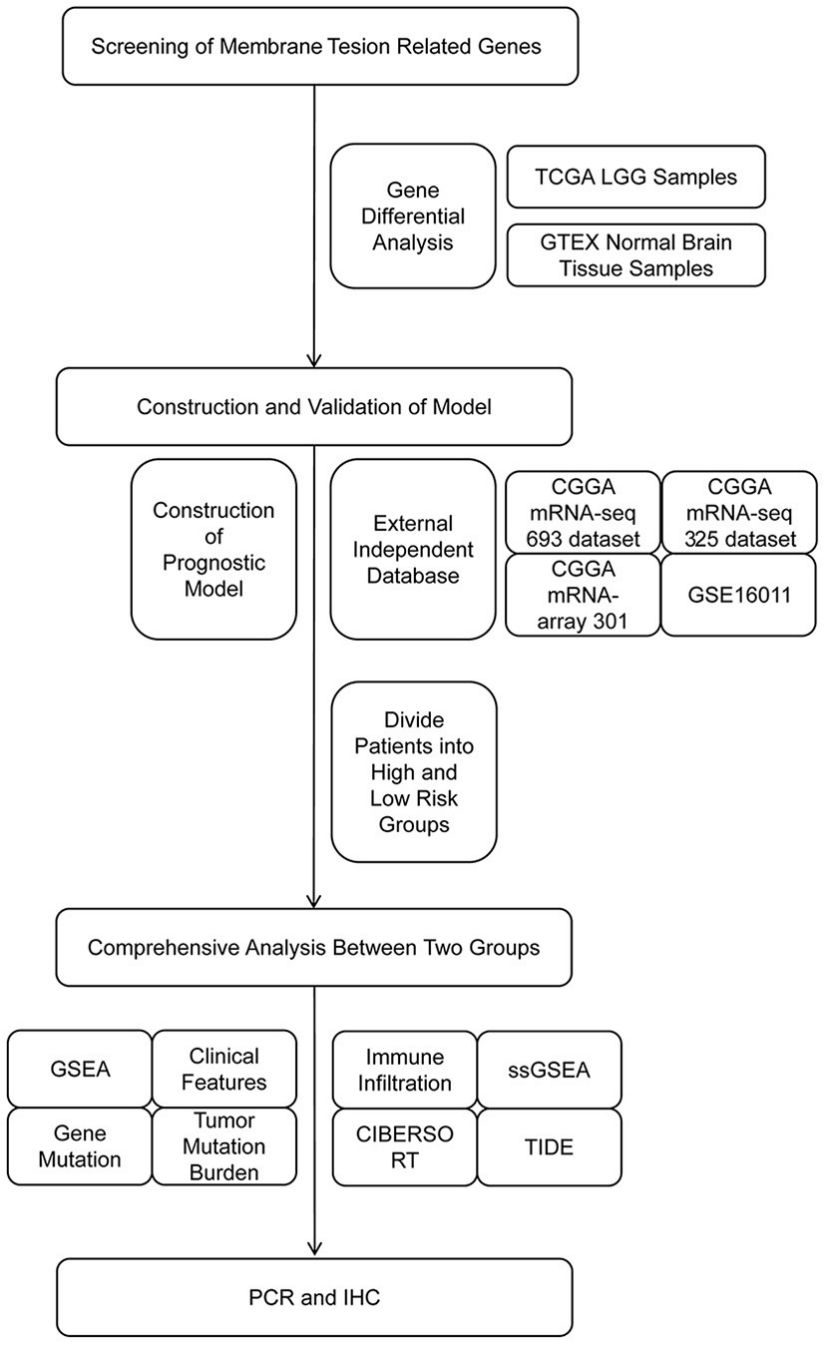
Flow diagram of the study.

The study flow diagram is presented in Figure 1. Collectively, we constructed and externally validated MTRG-based prognostic models which showed significant differential expression between LGG and normal brain tissues. We then performed gene enrichment, clinical characteristics, gene mutation, immune microenvironment and immunotherapy responsiveness analyses of patient groups according to the model. Finally, we validated our results through quantitative reverse-transcription polymerase chain reaction (qRT-PCR) and immunohistochemistry (IHC).

### Patient samples

RNA-seq data, survival information and baseline characteristics of 529 samples from patients with LGG were obtained from TCGA (https://tcga-data.nci.nih.gov/tcga/). RNA-seq data of 207 normal brain tissue samples were obtained from Genotype-Tissue Expression (GTEx, http://commonfund.nih.gov/GTEx/). Subsequently, mRNA expression data and survival information of GSE16011, mRNAseq_325, mRNAseq_693, mRNA-array_301 and Rembrandt microarray-independent datasets were downloaded from Gene Expression Omnibus (GEO, http://www.ncbi.nlm.nih.gov/geo) and China Glioma Genome Atlas (CGGA, https://www.cgga.org.cn/) for external validation. Gene mutation data were obtained from TCGA (15–18).

### Differential expression analysis

A total of 40 MTRGs were selected for expression analysis based on a literature search (19). The list of 40 MTRGs are included in Supplementary material 1. We performed differential expression analysis of MTRGs extracted from TCGA and compared them with the GTEx datasets. Differentially expressed genes were defined as those with a false discovery rate (FDR) *p* value of < 0.05 and absolute log2 fold change (logFC) of >1. We used the “limma” package in R for differential analysis, and normalized gene expression profiles using the normalizeBetweenArrays function (19). MTRGs with differential expression were uploaded to Metascape (http://metascape.org/) for functional analysis (20) and subsequently uploaded to String (https://string-db.org/) to construct a protein interaction network (21).

### Developing and validating our prognostic signature

To develop our prognostic signature, log-rank tests, LASSO regression and multivariate Cox regression were used to screen for differently expressed MTRGs that were significantly correlated with LGG prognosis. Multivariate Cox regression analysis was performed through a step function, and the lowest AIC value was selected as the final model. The risk score for each sample was calculated by multiplying the expression values of specific genes by their weights in the Cox model and adding them. We categorized the patients into low- and high-risk groups as per a median risk score. Kaplan–Meier (KM) curves were used to compare the OS between the two groups, and 3, 5, and 10-year receiver operating characteristic (ROC) curves were plotted. Area under curve (AUC) was used to evaluate the specificity and sensitivity of survival prediction. Finally, validation datasets were used to validate our prognostic signature.

### Comprehensive analysis of risk score

Patients were grouped according to different clinical characteristics (age: divided into younger and older age groups based on the median age; sex: female and male; grade: grades II and III; IDH1 mutation status: IDH1 mutant and IDH1 wild-type and 1p19q co-deletion status: 1p19q codel and 1p19q non-codel). The risk scores were analyzed separately for differences in the clinical features. Thereafter, in the gene mutation analysis, the “maftools” package in R was used to visualize the somatic mutations of the TCGA–LGG samples (22). We also calculated tumor mutational burden (TMB) for each LGG sample and compared the difference in TMB of the high- and low-risk groups. Finally, Spearman correlation was used to assess the association between TMB score and our risk score.

### Gene set enrichment analysis and related LncRNA exploration

Gene set enrichment analysis (GSEA) (23) was performed using GSEA v4.1.0 software (http://www.broadinstitute.org/gsea). The Hallmark v7.2, c2 KEGG, and c5 Go BP gene sets were used for further analyses (24). GSEA enrichment plots were constructed using the “ggplot2” package in R. In addition, long non-coding RNAs (lncRNAs) with a correlation coefficient of >0.5 and a *p* value of < 0.05 for ARFIP2, PICK1, SH3GL2, and SRGAP3 were considered to be significantly associated with our prognostic model. Subsequently, the lncRNA–mRNA network was constructed using Cytoscape 3.8.2, and univariate Cox regression was used to test the significance of the association between survival and lncRNAs (25).

### Immune characteristics and immune therapy analysis

Samples from the TCGA–LGG cohort were used for immune-related analysis. The enrichment scores of 16 immune cells and 13 immune-related functions per sample were estimated using single-sample GSEA (ssGSEA). The R package “CIBERSORT” was used to quantify 22 immune cell types. Only the samples with a CIBERSORT output *p* value of < 0.05 were selected for further analysis (26). We selected eight well-known glioma related immune checkpoints, obtained their mRNA expression and downloaded tumor immune dysfunction and exclusion (TIDE) scores, microsatellite instability (MSI), T cell dysfunction scores and T cell exclusion scores from the TIDE website (http://tide.dfci.harvard.edu) to explore the predictive ability of our prognostic model for immunotherapy response. All these immune-related characteristics of the high- and low-risk groups were compared. Finally, we compared the prognostic value among our prognostic signature, TIDE and T cell infiltration score (TIS) with the “timeROC” R package (27).

### Prognostic nomogram establishment

Using stepwise multivariate Cox regression analysis to screen prognostic factors, a model with a minimum AIC value used to assess the 3, 5, and 10-year OS of LGG, including age, grade, IDH mutation, 1p19q co-deletion and our risk score. Calibration curves were plotted to assess the predictive power of the nomogram prognostic model and the multivariate timeROC was plotted to compare the predictive power levels of various features.

### qRT-PCR and IHC

Total RNA was extracted using the TIANGEN RNA Extraction Kit (TIANGEN, Beijing, China) and reverse-transcribed using TOROIVD^®^ III Reverse Transcription Kit (Toroivd, Shanghai, China). Quantitative PCR was performed using TOROGreen qPCR Master Mix (Toroivd, Shanghai, China). The results are standardized using GAPDH. qPCR was performed using the Applied Biosystems QuantStudio Real Time PCR system and analyzed using Applied Biosystems QuantStudio Design & Analysis Software. Fold change was determined using the 2^−Δ*ΔCt*^ method for gene expression. Gene-specific PCR primers are listed in Supplementary material 2. IHC was performed according to the previously described protocol (28). LGG tissues were fixed with 4% paraformaldehyde overnight at 4°C. Subsequently, tissue samples were embedded in paraffin and sectioned at 4 μm. The sections were dewaxed with xylene, rehydrated using a graded series of alcohol and then placed in a retrieval box containing citrate antigen retrieval buffer (pH 6.0) for antigen retrieval in a microwave oven. Endogenous peroxidase activity was blocked by incubating with 3% H_2_O_2_ solution for 10 min. The sections were then blocked with 5% BSA for 30 min and incubated with SH3GL2 antibody (1:400, 12345-1-AP; ProteinTech) SRGAP3 antibody (1:200, ab204408; Abcam) overnight at 4°C in a humidified chamber. The sections were then incubated with biotinylated secondary antibody for 1 h and treated with SABC for 1 h. After each incubation step, sections were washed three times with phosphate-buffered saline for 5 min each. Finally, the specimens were stained with DAB chromogen (Zhongshan Jinqiao) and counterstained with hematoxylin. Immunohistochemical staining was performed using an immunohistochemical kit (Dako, Glostrup, Denmark). Clinical information on samples in qRT-PCR and IHC experiments is provided in Supplementary material 3. Immunohistochemical image analysis was conducted as follows: H-SCORE = ∑ (pi × i) = (percentage of weak intensity × 1) + (percentage of moderate intensity × 2) + (percentage of strong intensity × 3) (29, 30).

### Statistical analyses

Differences in the indicators of the high- and low-risk groups were compared by Wilcoxon test. Univariate survival analysis was performed by KM survival analysis and log-rank test. Multivariate survival analysis was performed using Cox regression models. Two-sided *p* < 0.05 was considered significant. Statistical data analysis was performed using the R software (Version 4.0.5).

## Results

### Differentially expressed MTRGs

**Figure 2:**
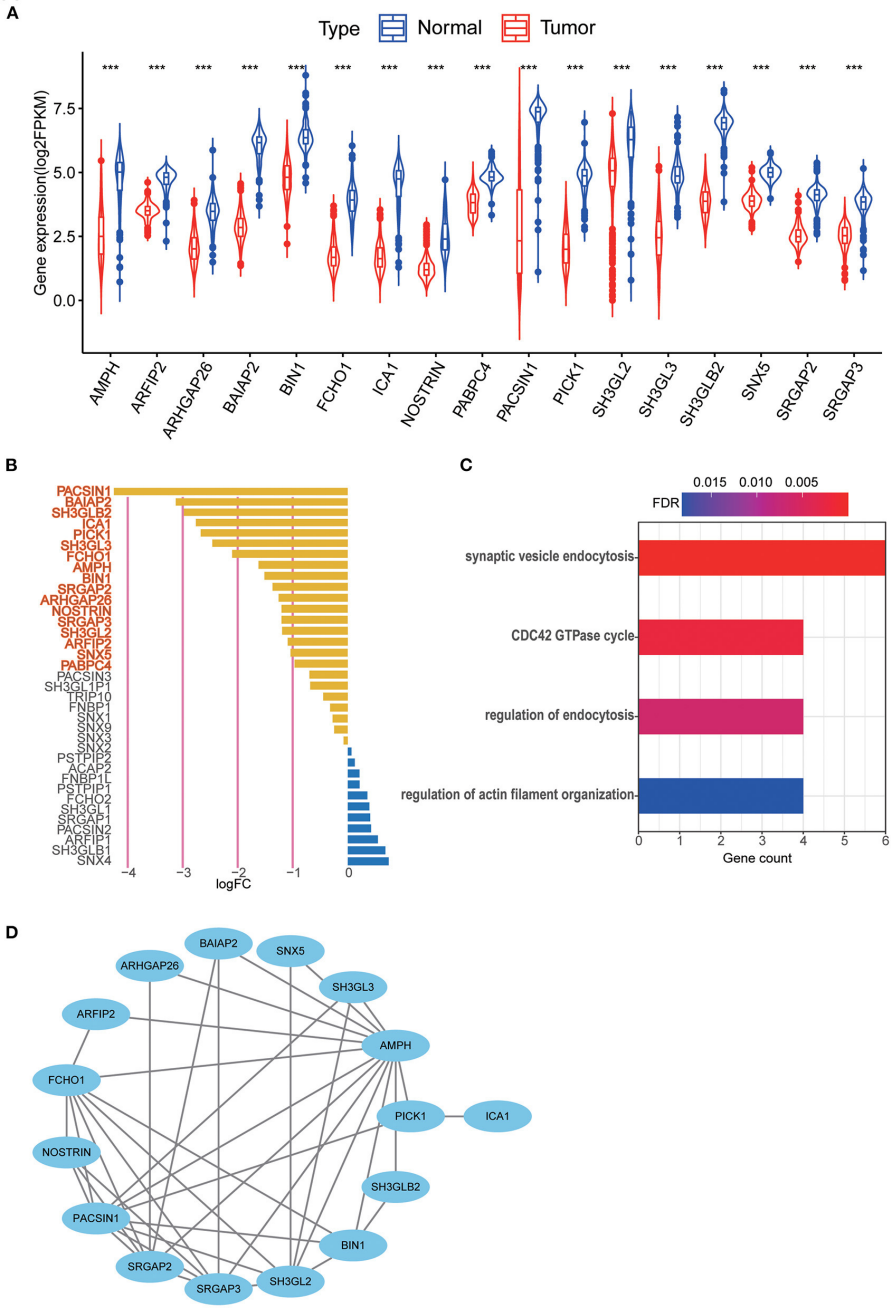
**(A)** 17 MTRGs with significantly different expression between LGG tissue and normal tissue, ***p < 0.001. **(B)** Significantly different expressed genes were selected through absolute log2FC > 1, FDR < 0.05. Selected genes are in bold. **(C)** Functional analysis of 17 MTRGs in Metascape. **(D)** PPI of significantly differential expressed genes.

The results of differential gene expression analysis of 40 MTRGs between 529 TCGA–LGG samples and 207 GTEx normal brain tissue samples are shown in Supplementary Figure 1A. Overall, 17 MTRGs were identified as differentially expressed genes (Figure 2A) and were ranked according to the significance of differences (Figure 2B). The results of functional analysis showed that the differentially expressed MTRGs in LGG and normal brain tissues were mainly enriched through synaptic vesicle endocytosis, CDC42 GTPase cycle, endocytosis regulation and actin filament organization regulation (Figure 2C). Finally, the protein–protein interaction network is shown in Figure 2D.

### Construction and validation of MTRG-related prognostic signature

**Figure 3:**
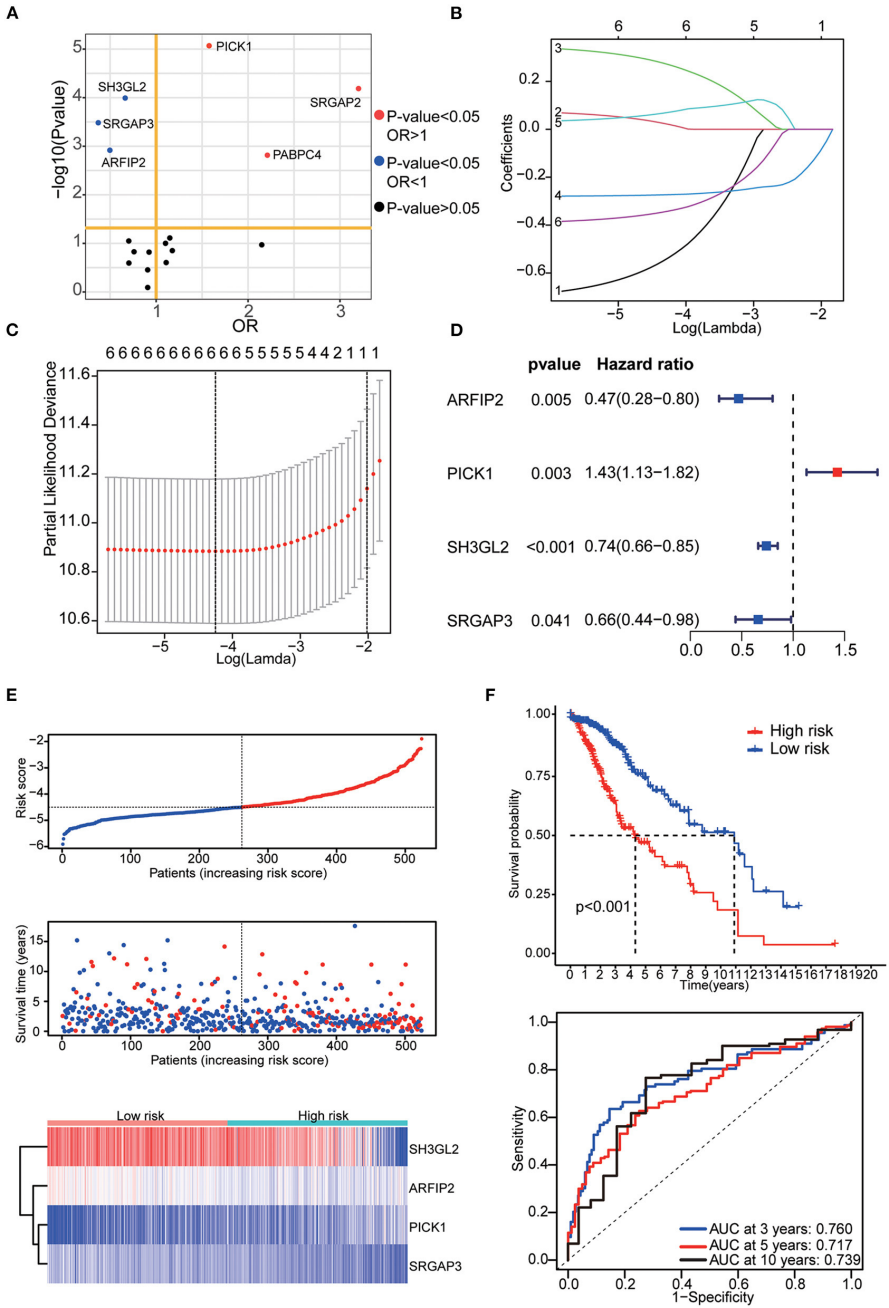
**(A)** Results of the KM curve analysis of 17 differential expressed MTRGs. Red dots represent the genes with an impact on poor prognosis, blue dots represent genes with an impact on good prognosis, and black dots represent genes with no significant impact on prognosis. **(B)** Coefficients of the determined characteristics are exhibited *via* lambda parameters. **(C)** Partial probability deviance relative to log (lambda) generated through LASSO and Cox regression. **(D)** Hazards ratio, *p* value and 95% confidence interval in stepwise multivariate Cox regression. **(E)** Riskscore, survival time and survival status in the TCGA–LGG data set. Above: The dotted line represents the median risk score, categorizing patients into low- and high-risk groups, with a scatter plot of Riskscore from low to high. Middle: The dotted line represents the median risk score, with the patients in the low-risk group on the left and those in the high-risk group on the right; the red dots represent dead patients, and the blue dots represent surviving patients. The patients in the high-risk group had shorter survival duration with a high mortality rate. Bottom: Heatmap illustrating the expression patterns of prognostic genes in low- and high-risk groups. PICK1 was more highly expressed in the high-risk group, whereas ARFIP2, SH3GL2, and SRGAP3 were more highly expressed in the low-risk group. **(F)** KM curve analysis of two groups and ROC curves for 3, 5, and 10-year OS.

The prognostic signature was constructed based on RNA-seq data from TCGA. Among 17 differentially expressed MTRGs, six of them had statistical significance in the prognosis of LGG using log-rank test. High expression of PICK1, SRGAP2 and PABPC4 were considered predictors of poor prognosis, whereas high SH3GL2, SRGAP3, and ARFIP2 expression were considered predictors of positive prognosis in LGG (Figure 3A). Using LASSO and stepwise multivariate Cox regression, we found that only four MTRGs—ARFIP2, PICK1, SH3GL2, and SRGAP3—significantly affected the OS of patients with LGG (Figures 3B,C). Next, we constructed a prognostic signature for all LGG samples and calculated their risk score formula as follows: −0.7459 × expression of ARFIP2 + 0.3572 × expression of PICK1 −0.2948 × expression of SH3GL2 −0.4229 × expression of SRGAP3 (Figure 3D). There were significant differences in terms of patient survival time, survival status and expression of the four MTRGs between the high- and low-risk groups (Figure 3E, Supplementary Figure 1B). The KM curve showed that the OS of the two groups was significantly different and the survival time of the high-risk group was significantly shorter than that of the low-risk group (*p* < 0.001). Finally, time-dependent ROC analysis revealed that the diagnostic accuracy is as follows: 3-year AUC = 0.760, 5-year AUC = 0.717 and 10-year AUC = 0.739 (Figure 3F).

**Figure 4:**
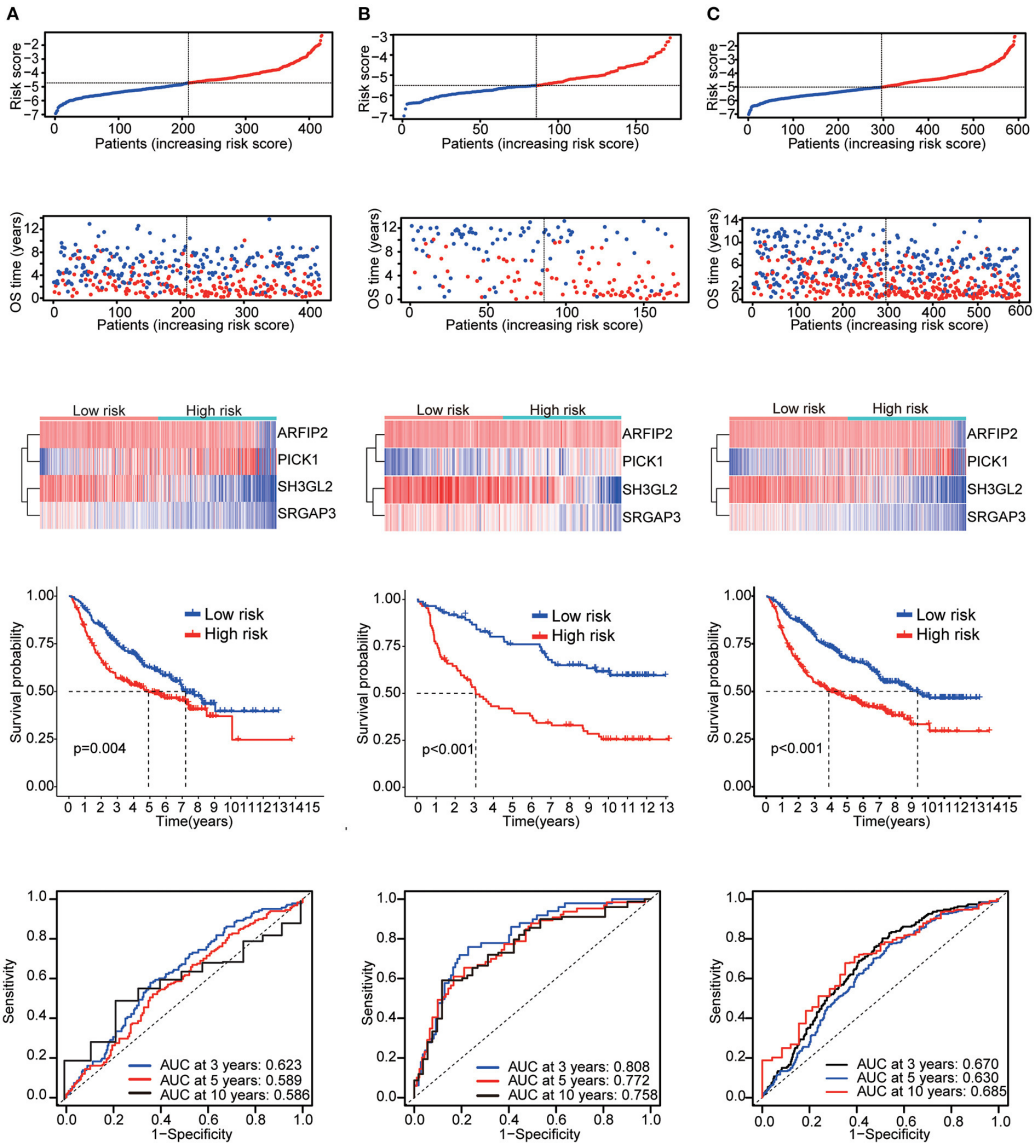
Validation results **(A)** CGGA mRNA-seq_693 cohort. **(B)** CGGA mRNAseq_325 cohort. **(C)** Whole CGGA mRNAseq cohort.

**Figure 5:**
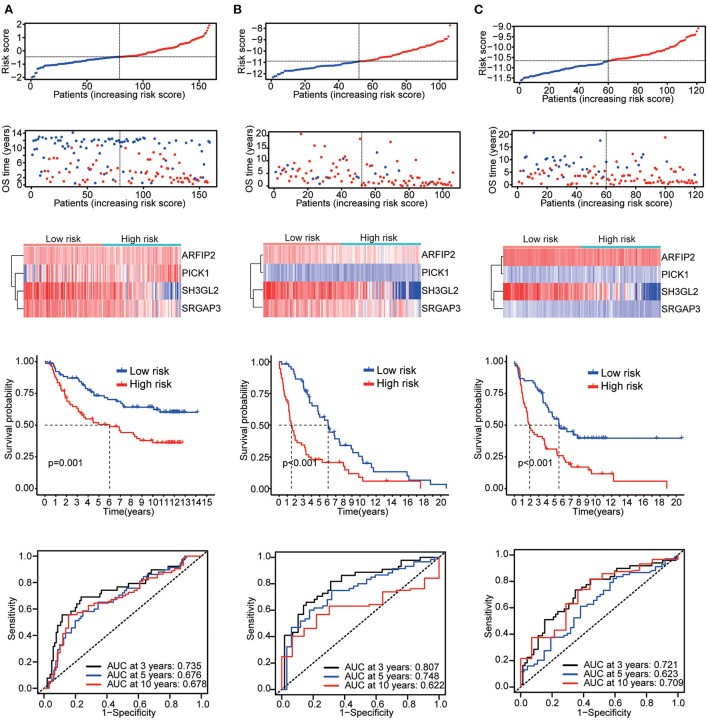
Validation results **(A)** CGGA array_301 cohort. **(B)** Rembrandt microarray cohort. **(C)** GSE16011 cohort.

Six validation datasets were used, including the CGGA mRNAseq_693, CGGA mRNAseq_325, the whole CGGA mRNAseq, CGGA mRNA array_301, Rembrandt microarray and the GSE16011 dataset to validate the prognostic model. Patients in each validation dataset were also divided into two groups according to their median risk score. There were also significant differences in the expression of the four genes between the high- and low-risk groups. KM curves and time-correlated ROC analyses were performed for six validation datasets. The results showed that our prognostic signatures could differentiate well between high- and low-risk populations. It could also effectively estimate OS probabilities for 3, 5, and 10 years (Figures 4, 5). The expression patterns of the four MTRGs between the high- and low-risk groups in the model are presented in Supplementary Figure 2.

### Clinical features and gene mutation landscape

**Figure 6:**
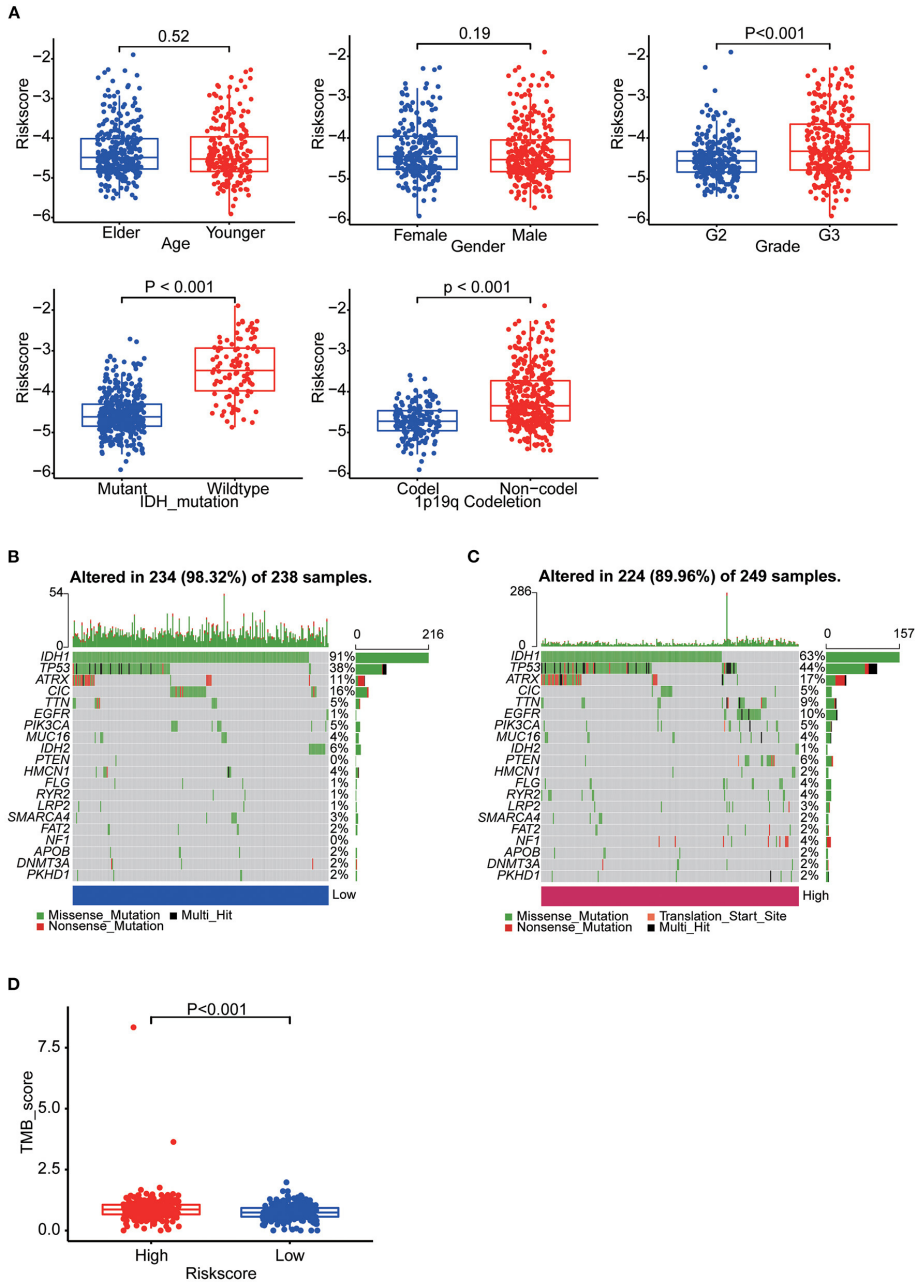
**(A)** Boxplots showing the distribution of risk scores in LGG samples categorized by age, sex, WHO grade, IDH mutation status and Chr1p19q co-deletion status. **(B)** The somatic landscape of low-risk group samples. **(C)** The somatic landscape of high-risk group samples. Mutation information for each gene in each sample is shown as waterfall plots, with different colors at the bottom with specific annotations indicating various mutation types. **(D)** Boxplot showing the difference of TMB scores between two groups.

Patients in TCGA were stratified as per their median age, sex, grade, IDH mutation and 1p19q co-deletion status, respectively. There were significant differences in risk scores among patients with different IDH mutation status, different tumor grades and different 1p19q deletion status. WHO grade III patients (*p* < 0.001), IDH-wild-type patients (*p* < 0.001) and chromosome 1p19q non-co-deletion patients (*p* < 0.001) had higher risk scores (Figure 6A). Differences in these molecular signatures suggested a strong relationship between risk scores and molecular and pathological tumor subtypes.

Epigenetic alteration plays a vital role in early malignancies and can influence prognosis. Here we obtained the mutation spectra of all LGG samples in TCGA. Missense mutations were the most common, followed by nonsense mutations. The most commonly mutated genes were *IDH1, IDH2, TP53, ATRX, EGFR, PTEN, CIC* and *TTN*. The results showed that mutations in *IDH1* (91%: 63%, *p* < 0.001) and *IDH2* (6%: 1%, *p* = 0.004) was more common in the low-risk group and mutations in *TP53* (44%: 38%, *p* = 0.177), *EGFR* (10%: 1%, *p* < 0.001) and *PTEN* (6%: 0%, *p* < 0.001) was more common in the high-risk group (Figures 6B,C). Furthermore, we calculated the TMB of each sample. Then, the TMB of the high- and low-risk groups was compared and the correlation between the risk score and the TMB score was analyzed. The results indicated the high-risk group had a higher TMB score (*p* < 0.001) and it was positively correlated with TMB (R = 0.22, *p* < 0.001) (Figure 6D, Supplementary Figure 3A).

### GSEA and MTRG-related lncRNA network

**Figure 7:**
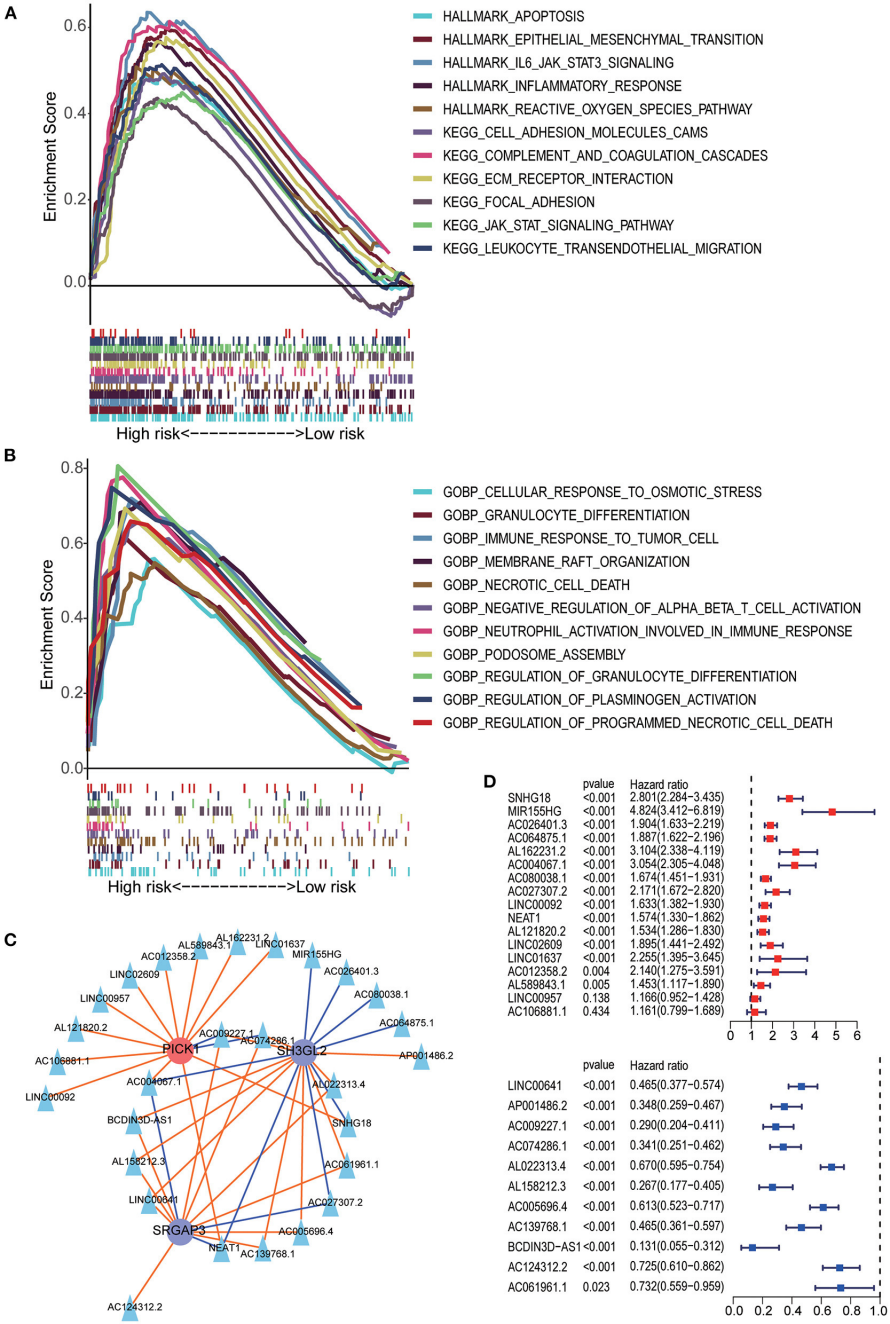
GSEA analysis between the high- and low-risk groups. **(A)** HALLMARK and KEGG pathway enrichment analysis. **(B)** GO BP enrichment analysis. **(C)** The lncRNA–MTRG regulation network; triangular nodes represent lncRNA, circular nodes represent prognostic MTRG, red circular nodes represent cancer-promoting genes, blue circular nodes represent tumor suppressor genes, red lines indicate upregulation and blue lines indicate downregulation. **(D)** Univariate Cox regression analysis of lncRNAs negatively correlated with SH3GL2 or SRGAP3 and positively correlated with PICK1. Univariate Cox regression analysis of lncRNAs positively correlated with SH3GL2 or SRGAP3 and negatively correlated with PICK1.

The results of GSEA showed that the apoptosis pathways, immune response related pathways and processes, cell endocytosis, exocytosis and cell adhesion biology processes was enriched in the high-risk group. Detailed results of GSEA are shown in Figures 7A,B.

Previous studies have shown that lncRNAs can regulate the expression of target genes in their cis or trans forms, which is the main regulatory mechanism of lncRNAs (31–34). To explore the possible regulatory mechanisms of MTRGs in our prognostic model for LGG, we screened 28 highly correlated lncRNAs and constructed a lncRNA–mRNA regulatory network (Figure 7C). In our prognostic signature, high expression of SH3GL2 and SRGAP3 suggested a beneficial effect on survival, but high expression of PICK1 led to a poor survival rate. Those lncRNA which positively regulated SH3GL2 or SRGAP3 or negatively regulated PICK1 might benefit in terms of survival. Conversely, the other lncRNAs might have a deleterious effect on patient survival. Survival analysis of the 28 lncRNAs was performed using the KM curve, and the results showed that only two of them had no significant effect on survival; 26 of 28 lncRNAs were consistent with the expected results (Figure 7D). NEAT1, AC004067.1, AC009227.1, and AC074286.1 were associated with all three MTRGs. Among them, NEAT1 and AC004067.1 were not only positively correlated with SH3GL2 and SRGAP3 but negatively correlated with PICK1, whereas AC009227.1 and AC074286.1 were negatively correlated with SH3GL2 and SRGAP3 and positively correlated with PICK1. Subsequent studies should explore whether these four lncRNAs have a binding relationship with MTRGs.

### Immune-correlation and immunotherapy benefit analysis

**Figure 8:**
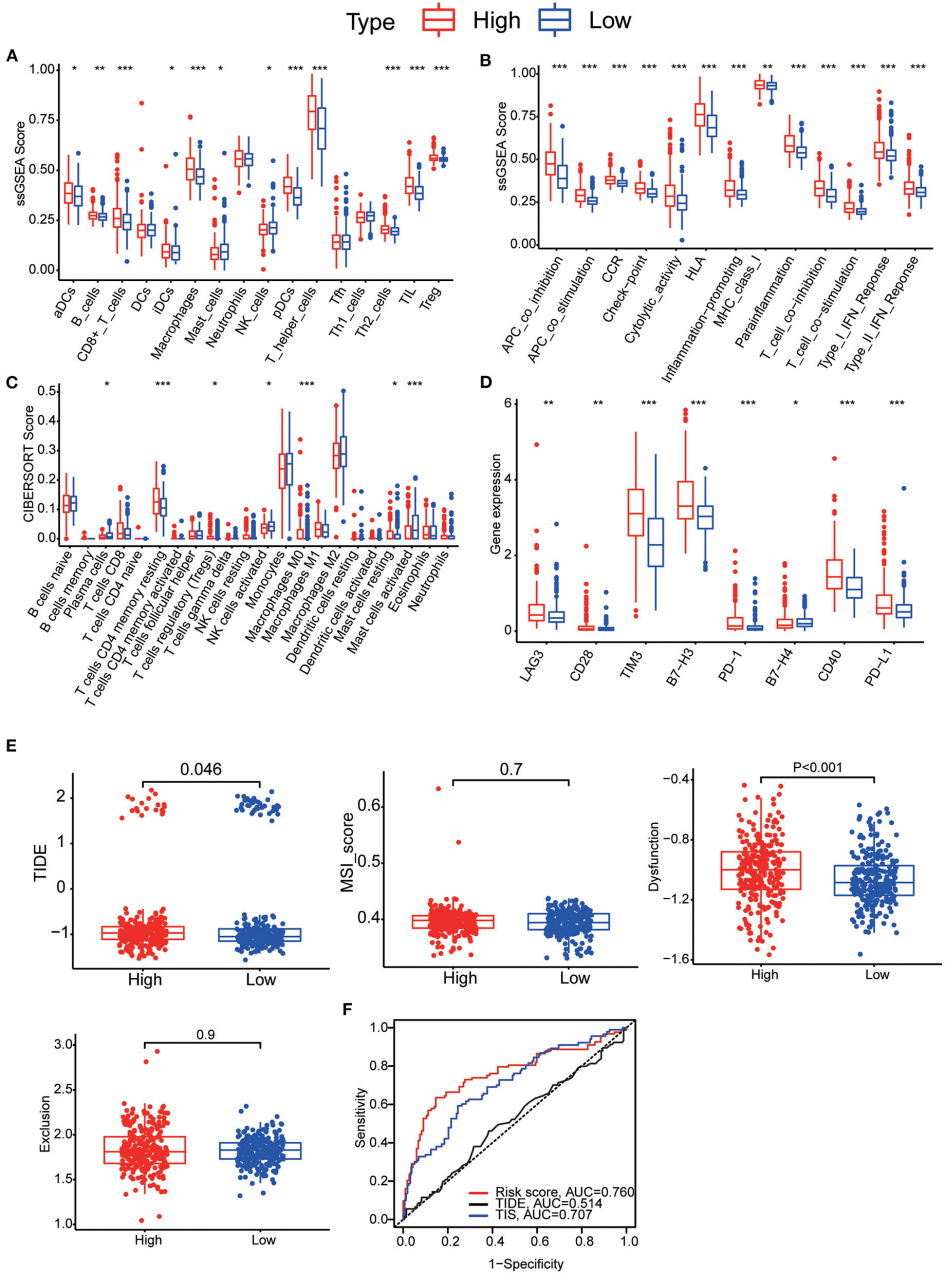
**(A)** Boxplots illustrating the 16 immune cells in the two groups using ssGSEA analysis. **(B)** Boxplots illustrating the 13 immune-linked functions in two groups using ssGSEA analysis. **(C)** Boxplots illustrating the level of 12 immune cells infiltration in two groups using CIBERSORT analysis. **(D)** Boxplots illustrating the expression of immune check point genes in high- and low-risk groups. **(E)** TIDE, MSI and T cell dysfunction and exclusion score in two groups. The variables between the two groups were compared through the Wilcoxon test, * *p* < 0.05, ** *p* < 0.01, *** *p* < 0.001. **(F)** Time-dependent ROC was used to compare the prediction performance of three prognostic models (our MTRGs related risk score, TIDE, TIS).

To further explore the correlation between the 11-gene signature risk score and immune status, we used ssGSEA to quantify the enrichment scores of 16 immune cell subgroups and 13 related functions, or pathways. Overall, 12 types of immune cells were significantly different between high- and low-risk groups (Figure 8A). Likewise, all 13 immune-related functions were significantly different between the two groups, which revealed a significant relationship with risk scores (Figure 8B). Then, CIBERSORT was used to calculate the abundance of 22 immune cells. We used the Wilcoxon test to compare the distribution of immune cells in different risk subgroups. We found that resting CD4 memory T cells, regulatory T cells, M0 macrophages and resting mast cells were more abundant in the high-risk subgroup, whereas plasma cells, activated NK cells and activated mast cells were more abundant in the low-risk subgroup (Figure 8C). We found that immune checkpoints (PD-1, PD-L1, LAG3, B7-H3, B7-H4, CD28, CD40, and TIM3) were significantly highly expressed in the high-risk group (Figure 8D). We also found higher TIDE scores (*p* = 0.046) and dysfunction scores (*p* < 0.001) in the high-risk group than in the low-risk group, suggesting that the low-risk group would benefit more from immune checkpoint inhibitor (ICI) therapy (Figure 8E). Finally, we calculated the AUC for TIDE = 0.514 and for T cell-inflamed signature (TIS) = 0.707, so we suggest that the predictive value of our prognostic signature was better than TIS and TIDE in LGG (Figure 8F).

### Construction of nomogram

**Figure 9:**
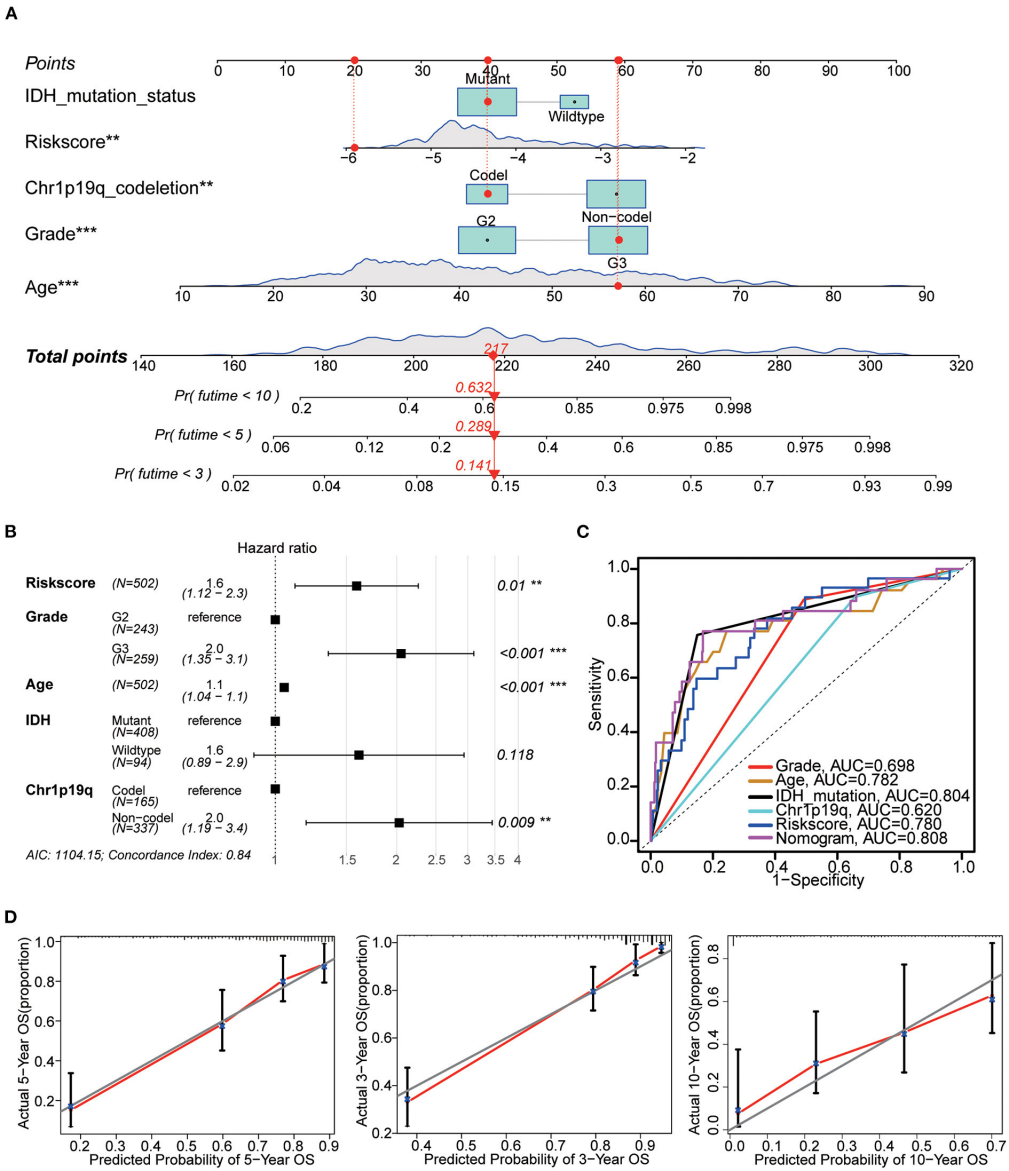
Nomogram construction. **(A)** The nomogram used risk score, age, grade, IDH mutation and 1p19q co-deletion to assess the 3, 5, and 10-year overall survival of LGG. **(B)** Hazards ratio, *p* value and 95% CI of each variable in the nomogram model. Model's AIC value and c-index are at the bottom. **(C)** Multivariate ROC analysis was used to compare the predictive power of each variable. Among them, the nomogram has the best predictive ability. **(D)** The calibration curve for the evaluation of the nomogram. The Y-axis designates the actual survival, while the X-axis designates nomogram estimated 3, 5, and 10-year OS of patients in TCGA cohort. **p < 0.01, ***p < 0.001.

The nomogram included the risk score and baseline characteristics (age, grade, IDH mutation status and 1p19q co-deletion status) and was built to estimate 3, 5, and 10-year survival probabilities (Figures 9A,B). We found that the AUC for OS from the nomogram model was greater than any other factor, implying that the nomogram incorporated risk score and clinical features could assess patient survival more accurately than a single prognostic signature (Figure 9C). For calibration curves forecasting 3, 5, and 10-year survival, red lines represent the estimated survival and gray lines represent the ideal survival time. All three lines were tightly aligned, showing good calibration in the TCGA dataset (Figure 9D). Data from the validation sets (CGGA mRNAseq 693 and CGGA mRNA-seq 325) were also acceptable in terms of predictive power (Supplementary Figure 3B).

### Validation of MTRGs expression levels in the prognostic model

**Figure 10:**
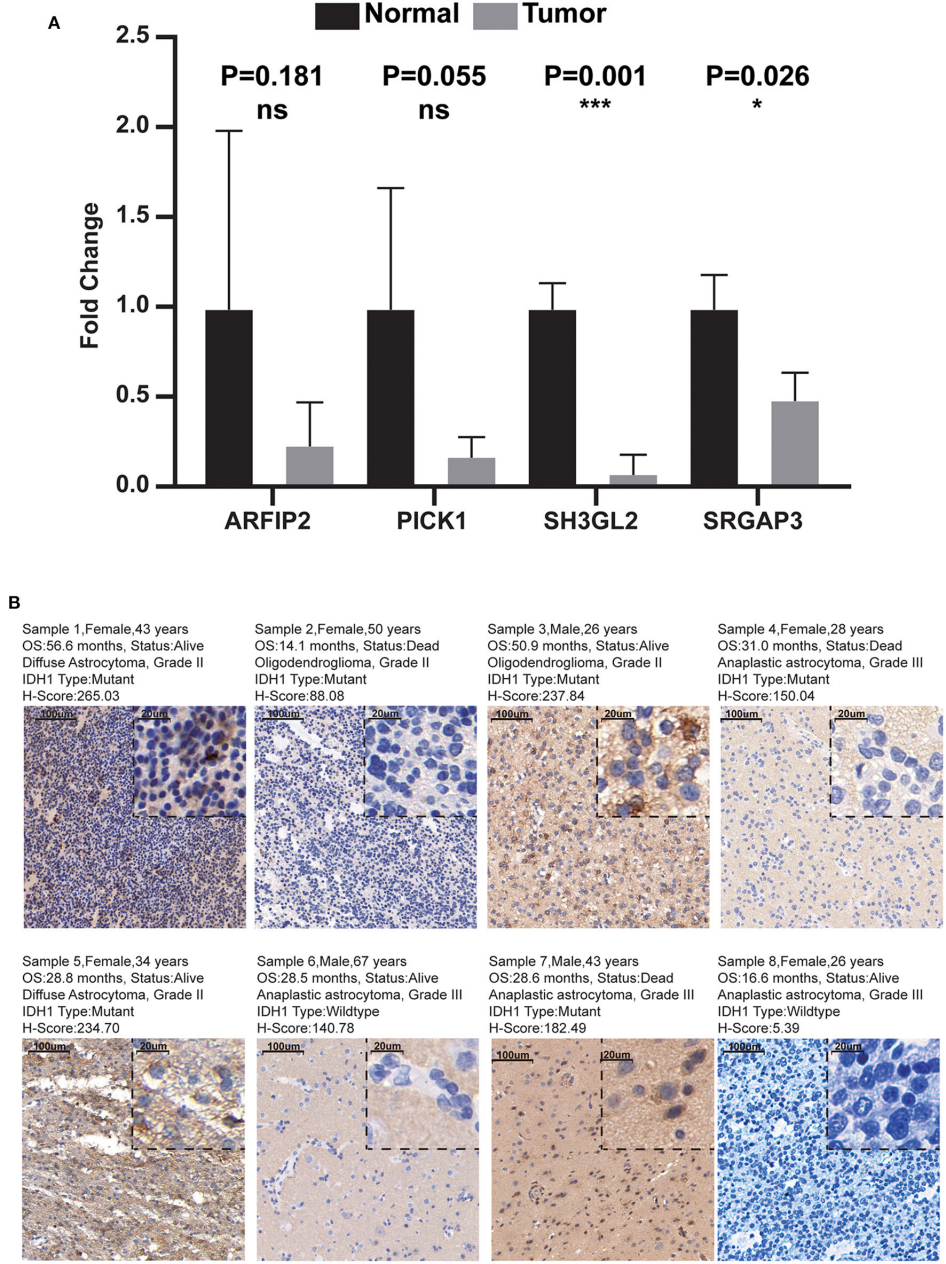
Results of qRT-PCR and IHC. **(A)** Expression distribution of risk score MTRGs in normal brain and glioma samples. Asterisks represent level of significance *p ≤ 0.05, ***, *p* ≤ 0.001, ns, not statistically significant. **(B)** Immunohistochemical results of eight glioma samples (magnification, 10× and 40×).

Combined with the results of qRT-PCR, we noticed that the RNA expression levels of ARFIP2, PICK1, SH3GL2, and SRGAP3 in normal brain tissue were higher than those in LGG tissue, and the difference between SH3GL2 and SRGAP3 was statistically significant (Figure 10A). In addition, the IHC results showed that samples from two patients with a survival period of more than 50 months had high expression of SH3GL2 (Samples 1 and 3). The samples from three deceased patients revealed low SH3GL2 expression (Samples 2, 4 and 7). SH3GL2 protein expression was low in two IDH1 wild-type samples (Samples 6 and 8), one of which hardly expressed SH3GL2 protein (Figure 10B). In summary, SH3GL2 is at the RNA and protein levels. In summary, SH3GL2 showed significant inter-group differences in expression at both RNA and protein levels. The immunohistochemical results of SRGAP3 are shown in Supplementary Figure 4, and they revealed that SRGAP3 was not expressed in three of the four samples.

## Discussion

Gliomas are common aggressive primary malignant brain tumors that occur in adults. Current treatment strategies for glioma are not adequately effective because of their high infiltration rate and heterogeneity. Even after comprehensive treatment, gliomas are prone to recurrence and can easily invade surrounding normal tissues (1–4, 35). Recently, increasing evidence indicates that cellular mechanics are intrinsically linked to cancer invasion and metastasis. High membrane tension helps maintain cell integrity and increases cell stiffness, thereby inhibiting cell migration (7–9, 36, 37). Malignant cells are mechanically characterized by reduced cell stiffness, which is strongly correlated with increased invasiveness and metastasis (10–12, 38, 39).

To the best of our knowledge, the prognostic value of plasma MTRGs in LGG is yet to be elucidated. In this study, we systematically analyzed the differences in the expression of 40 MTRGs in tumor tissues and normal brain tissues and examined their relationship with patient prognosis. We then constructed an MTRG-based prognostic risk score model using the TCGA–LGG cohort and validated its stability and effectiveness in the CGGA, GEO, and REMBRANT cohorts. Based on this proposed model, the TCGA–LGG cohort was divided into high- and low-risk groups for further analysis. The results demonstrated that patients with poorer pathological grades had higher risk scores. Other factors, including IDH1/2, TP53 and EGFR mutation status and 1p19q co-deletion status, which are considered to be associated with glioma progression and patient prognosis, are also significantly different for high- and low-risk groups. These results are consistent with those of previous prognostic models that revealed that high-risk scores are inversely associated with patient outcomes. To further explore the signaling pathways associated with prognostic models, we performed GSEA and noted that our model was associated with multiple cancer-related pathways, including apoptosis, epithelial–mesenchymal transition, reactive oxygen species and immune-related pathways. Furthermore, we constructed an lncRNA–mRNA network to provide potentially useful information on lncRNA–mRNA interaction mechanisms in LGG progression.

Of the four genes, ARFIP2, SH3GL2, and SRGAP3 have a protective function. ARFIP2 is a member of the BAR family and plays a role in constitutive metalloproteinase (MMP) secretion from the trans-Golgi network. It is also involved in autophagy through the regulating of ATG9A vesicles that deliver phosphatidylinositol 4-kinase beta to the autophagosome initiation site (40). SH3GL2, also known as endophilin-A1, is involved in synaptic vesicle endocytosis and may recruit other proteins to membranes with high curvature (41). Zhu et al. found that SH3GL2 inhibits the migration and invasion behavior of glioma cells by negatively regulating STAT3/MMP2 signaling. Moreover, SH3GL2 dysregulation has been associated with increased blood–brain barrier permeability, which may promote tumor progression (42). SRGAP3 regulates the actin cytoskeleton by inhibiting actin dynamics specifically via lamellipodia formation (43). High expression of ARFIP2, SH3GL2, and SRGAP3 help maintain cell tension and stiffness as well as inhibit tumor invasion and metastasis. Finally, PICK1, a protein containing PDZ and BAR domains, inhibits actin polymerisation dependent on actin-related protein 2/3 and is involved in regulating the trafficking of many cell surface receptors (44). Experimental evidence suggests that PICK1 is involved in promoting tumor growth and is associated with poor prognosis in human breast cancer (45). In conclusion, our risk scores were negatively correlated with the expression levels of ARFIP2, SH3GL2, and SRGAP3, whereas the risk scores were positively correlated with the expression level of PICK1. Finally, qRT-PCR and IHC analysis confirmed the differential expression of SH3GL2 in tissues of different origin and its correlation with the prognosis of patients with glioma.

Recently, the most advanced therapy for cancer is immunotherapy. According to the correlation analysis between the proposed model and immune cell infiltration, immune-related activities using ssGSEA and CIBERSORT, our prognostic signature could reflect the tumor immune microenvironment. NK cell infiltration levels were significantly higher in the low-risk group, indicating greater antitumor ability. Subsequently after the TIDE analysis, it was found that the low-risk group had lower TIDE and immune dysfunction scores. These results suggest that patients in the low-risk group benefit more from ICI therapy than those in the high-risk group.

## Conclusion

Our prognostic signature was validated using six independent datasets and exhibited better predictive ability than TIDE and TIS scores. Our MTRGs related prognostic signature is, therefore, a promising predictive tool for better risk-stratification of patients with LGG.

## Data availability statement

The original contributions presented in the study are included in the article/Supplementary material, further inquiries can be directed to the corresponding author.

## Ethics statement

Ethical review and approval was not required for the study on human participants in accordance with the local legislation and institutional requirements. Written informed consent from the patients/participants or patients/participants' legal guardian/next of kin was not required to participate in this study in accordance with the national legislation and the institutional requirements.

## Author contributions

JL and TJ wrote the manuscript and designed the whole work. JL and FL collected and analyzed the raw data. FL helped to revise the manuscript. All authors contributed to the article and approved the submitted version.

## Funding

This research was supported by the Wenzhou Science and Technology Project under Grant No. Y20190144.

## Conflict of interest

The authors declare that the research was conducted in the absence of any commercial or financial relationships that could be construed as a potential conflict of interest.

## Publisher's note

All claims expressed in this article are solely those of the authors and do not necessarily represent those of their affiliated organizations, or those of the publisher, the editors and the reviewers. Any product that may be evaluated in this article, or claim that may be made by its manufacturer, is not guaranteed or endorsed by the publisher.

## Supplementary material

Supplementary Figure 1 Differential analysis of MTRGs. **(A)** Differential expression analysis of 40 MTRGs in LGG tumor samples (TCGA–LGG) and normal brain tissue samples (GTEx). **(B)** Differential expression analysis of four MTRGs in prognostic models between high- and low-risk groups in the TCGA–LGG dataset. ^*^*p* < 0.05, ^***^*p* < 0.001.

Supplementary Figure 2 Difference analysis of four MTRGs in prognostic models between high- and low-risk groups in the six validation datasets (CGGA_mRNAseq 325, CGGA_mRNAseq 693, CGGA_mRNAseq 325 + CGGA_mRNAseq 693, CGGA_microarray 301, Rembrandt and GSE16011). **p* < 0.05, ***p* < 0.01, and ****p* < 0.001.

Supplementary Figure 3 **(A)** Correlation of risk scores and tumor mutational burden in the TCGA–LGG cohort. **(B)** Calibration curves for 3, 5, and 10-year OS predicted in CGGA_mRNAseq 693 data set with nomogram. **(C)** Calibration curves for 3, 5, and 10-year OS predicted in CGGA_mRNAseq 325 data set with nomogram.

Supplementary Figure 4 IHC results of SRGAP3 in four samples (Sample 5, Sample 6, Sample 7, Sample 8). SRGAP3 was not expressed in Samples 5, 6 and 8 and moderately expressed in Sample 7.
