# Supplementary figures and images for: Structuring and validating a prognostic model for low-grade gliomas based on the genes for plasma membrane tension
Source: Front Neurol. 2022 Nov 3;13:1024869. doi: 10.3389/fneur.2022.1024869 (PMC9668894; doi:10.3389/fneur.2022.1024869)

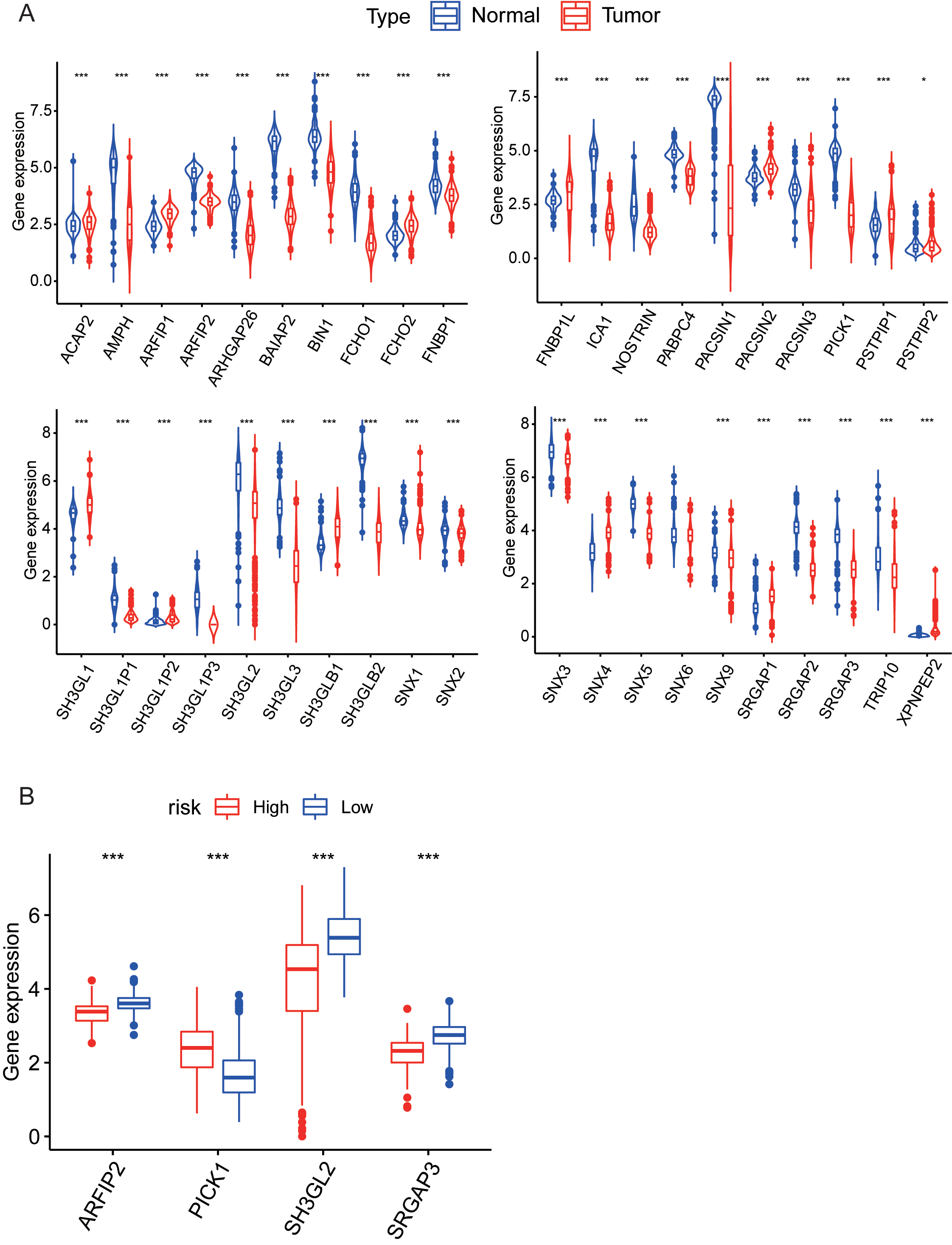

Supplement: Supplementary Figure 1 — Differential analysis of MTRGs. (A) Differential expression analysis of 40 MTRGs in LGG tumor samples (TCGA–LGG) and normal brain tissue samples (GTEx). (B) Differential expression analysis of four MTRGs in prognostic models between high- and low-risk groups in the TCGA–LGG dataset. *p < 0.05, ***p < 0.001. [file Image_1.TIF]

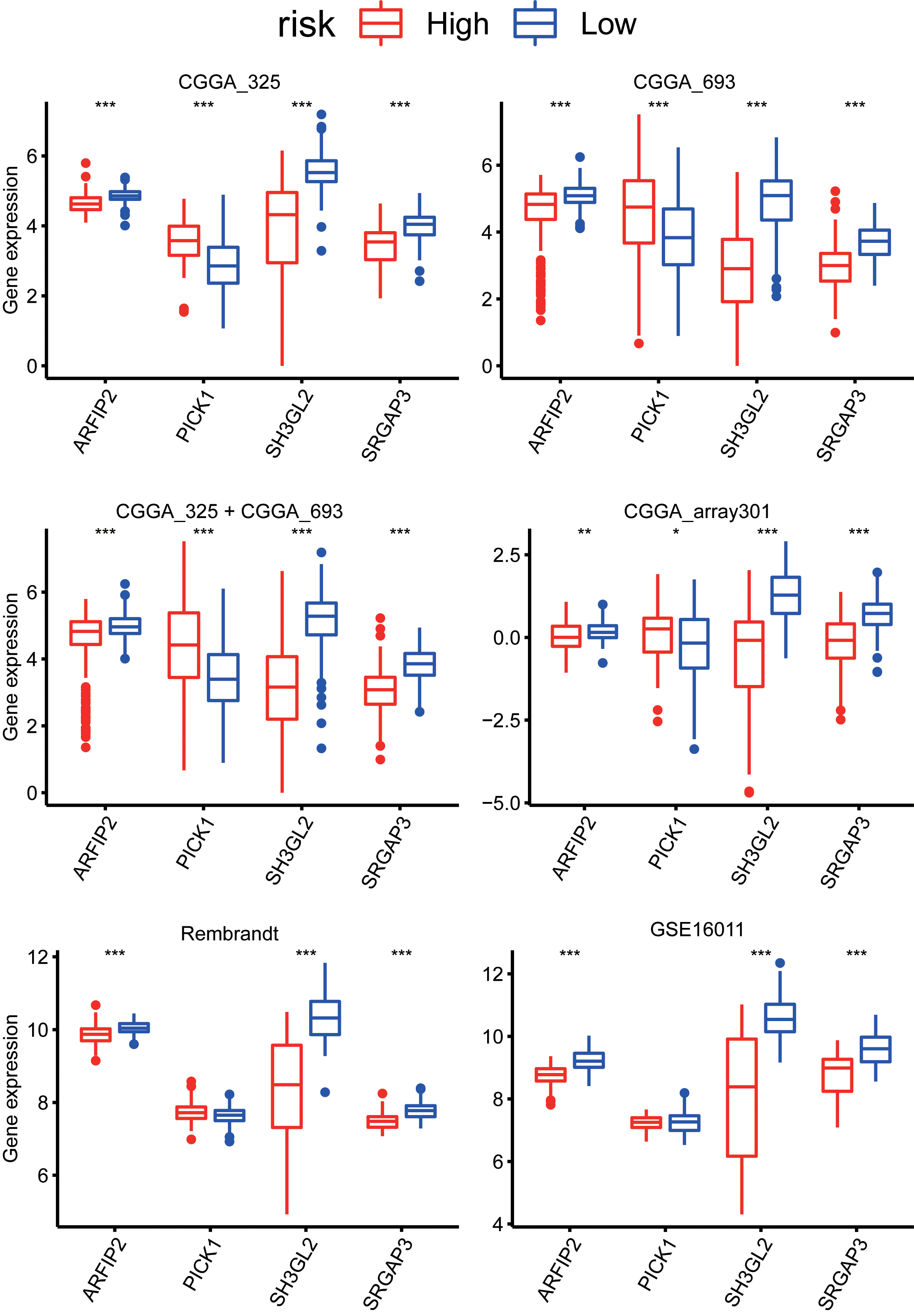

Supplement: Supplementary Figure 2 — Difference analysis of four MTRGs in prognostic models between high- and low-risk groups in the six validation datasets (CGGA_mRNAseq 325, CGGA_mRNAseq 693, CGGA_mRNAseq 325 + CGGA_mRNAseq 693, CGGA_microarray 301, Rembrandt and GSE16011). *p < 0.05, **p < 0.01, and ***p < 0.001. [file Image_2.TIF]

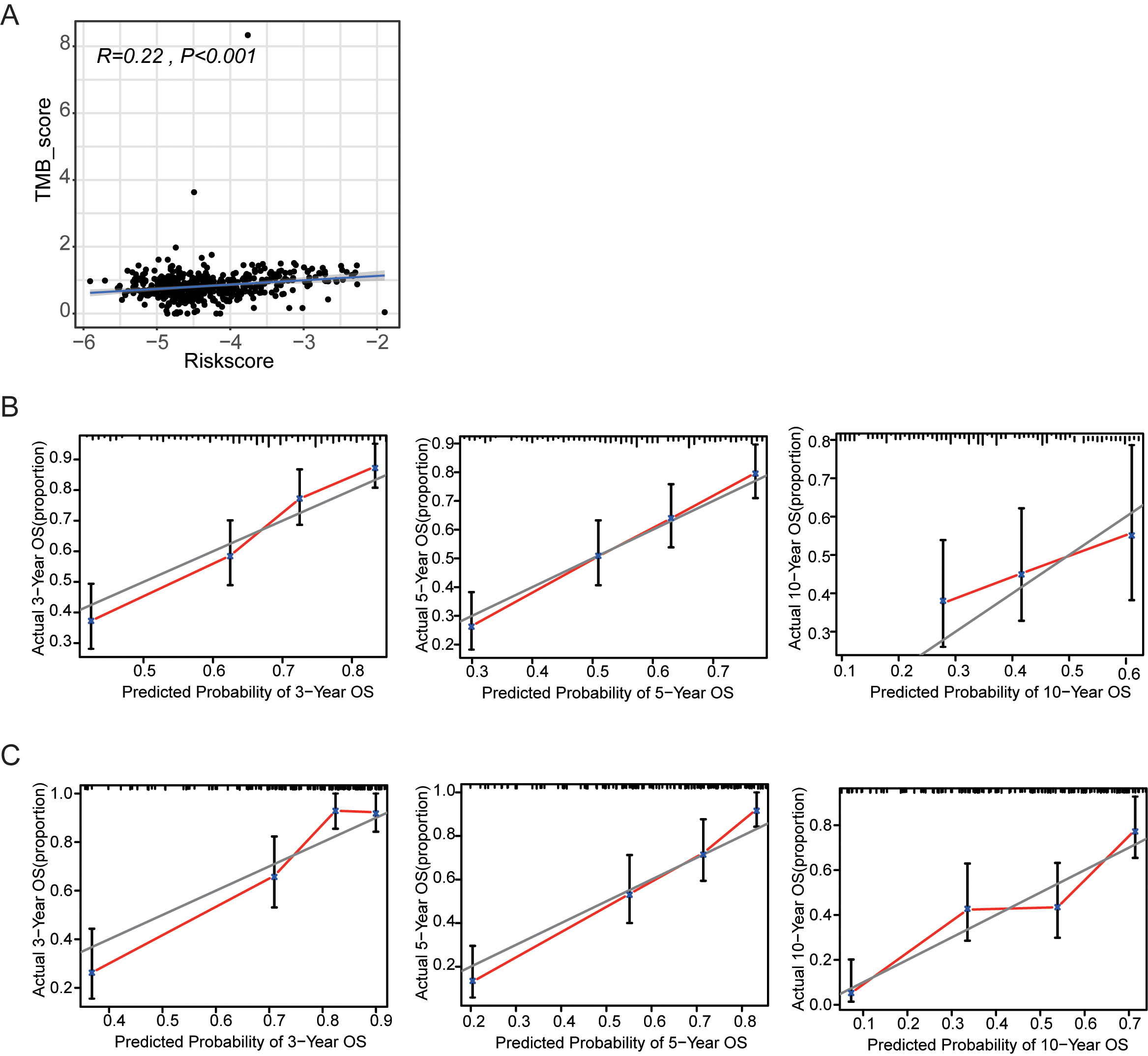

Supplement: Supplementary Figure 3 — (A) Correlation of risk scores and tumor mutational burden in the TCGA–LGG cohort. (B) Calibration curves for 3, 5, and 10-year OS predicted in CGGA_mRNAseq 693 data set with nomogram. (C) Calibration curves for 3, 5, and 10-year OS predicted in CGGA_mRNAseq 325 data set with nomogram. [file Image_3.TIF]

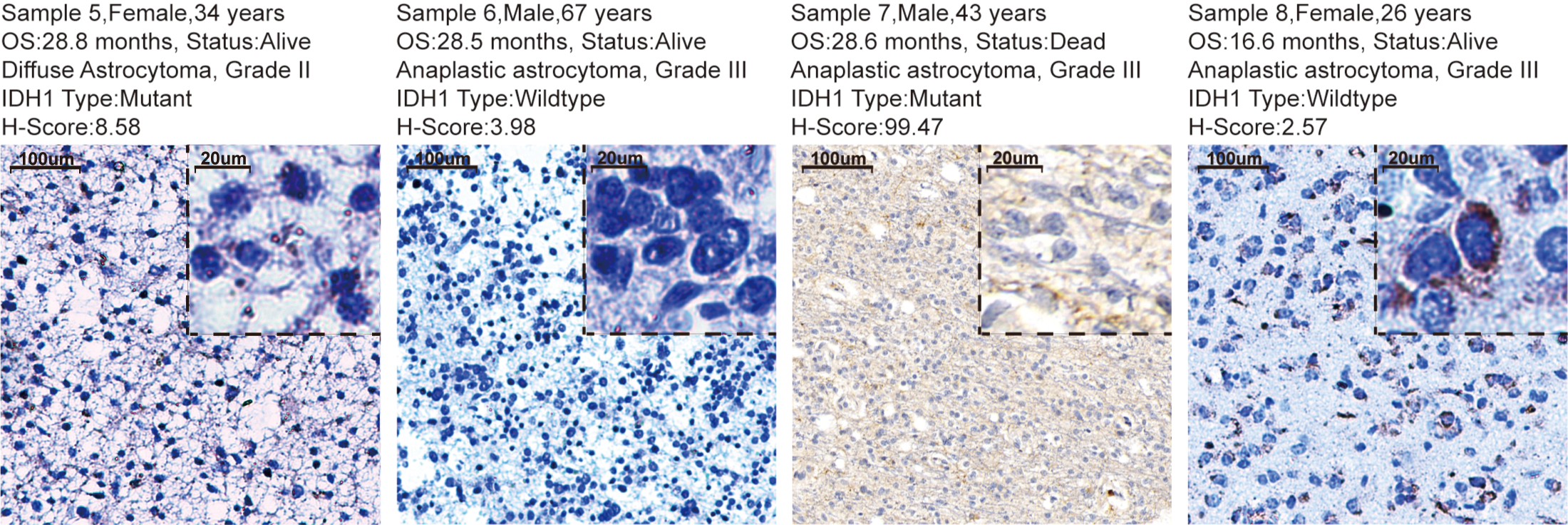

Supplement: Supplementary Figure 4 — IHC results of SRGAP3 in four samples (Sample 5, Sample 6, Sample 7, Sample 8). SRGAP3 was not expressed in Samples 5, 6 and 8 and moderately expressed in Sample 7. [file Image_4.TIF]
